# Simultaneous Reconstruction of Gas Concentration and Temperature Using Acoustic Tomography

**DOI:** 10.3390/s24103128

**Published:** 2024-05-14

**Authors:** Shuangling Liu, Ming Zhu, Meng Deng, Zesheng Hu, Zhuo Cheng, Xingshun He

**Affiliations:** 1 School of Mechanical Engineering and Electronic Information, China University of Geosciences, Wuhan 430079, China; d202080750@hust.edu.cn; 2 Hubei Key Laboratory of Smart Internet Technology, School of Electronic Information and Communications, Huazhong University of Science and Technology, Wuhan 430074, China; 3 Xi’an Modery Chemistry Research Institute, Xi’an 710065, China

**Keywords:** acoustic tomography, speed of sound dispersion, concentration field, temperature field

## Abstract

Acoustic tomography utilizes sensor arrays to collect sound wave signals, enabling non-contact measurement of physical parameters within an area of interest. Compared to optical technologies, acoustic tomography offers the advantages of low cost, low maintenance, and easy installation. Current research in acoustic tomography mainly focuses on reconstruction algorithms for temperature fields, while monitoring the composition and concentration of gases is significant for ensuring safety and improving efficiency, such as in scenarios like boiler furnaces and aviation engine nozzles. In excitable gases, the speed of sound exhibits an S-shaped curve that changes with frequency, a characteristic that could be potentially useful for acoustic tomography. Therefore, this study primarily discusses the quantitative calculation of gas concentration and temperature based on the dispersion of the speed of sound. By employing graphic processing and pattern matching methods, a coupled relationship of the dispersion of the speed of sound with gas concentration and temperature is established. The projection intersection method is used to calculate the concentration and temperature of binary and ternary gas mixtures. Combined with the inversion method, a joint reconstruction method for gas concentration fields and temperature fields based on the dispersion of the speed of sound is developed. The feasibility of the proposed simultaneous reconstruction method for temperature and concentration fields is validated using numerical simulations. Additionally, an acoustic tomography experimental system was set up to conduct reconstruction experiments for binary gas concentration fields and temperature fields, confirming the effectiveness of the proposed method.

## 1. Introduction

Gases are often produced in industrial processes, such as boiler furnaces and aviation engine nozzles, and they are closely related to system safety and production efficiency [1,2]. However, gases are often characterized as invisible and intangible, making it difficult for people to detect their presence. If it were possible to directly see the concentration field of gases, it would be highly beneficial for the measurement and monitoring of gases. Therefore, it is of great significance to rapidly and accurately obtain information about the composition of the gas in the target environment [3,4].

The state field of gases mainly includes concentration fields and temperature fields [5,6,7,8]. In the realm of tomography methods, optics and acoustics are two typical representatives. The optical method primarily involves tunable diode laser absorption spectroscopy (TDLAS) [9,10,11]. TDLAS is based on the characteristic spectral absorption of molecules. By measuring the attenuation of the laser power, spectral data like absorbance can be obtained, which allows for the quantitative measurement of gas concentration, temperature, and other parameters within the area. Although optical methods offer high measurement precision, they also have certain limitations: (1) Optical instruments are expensive and easily damaged; and (2) optical instruments require alignment, and their arrangement is relatively complex.

Compared to lasers that propagate in straight lines, sound waves can spread spherically, making sensor installation convenient and providing more propagation paths [12,13,14,15]. Acoustic sensors are cost-effective and durable, allowing for the placement of more measurement points with lower maintenance costs [16,17]. From a practical standpoint, acoustic tomography (AT) is more suitable for large-scale applications in gas imaging [18,19]. In recent years, with the popularization of products such as microphones, speakers, and ultrasonic transducers, people have begun to research measuring the state fields of gases with sound waves [20,21].

However, recent research on AT has mainly focused on the measurement of only the temperature field, but there is a paucity of studies on the simultaneous reconstruction of the temperature and concentration fields. Only Liu Yan proposed a method to reconstruct the concentration field, temperature field, and velocity field by measuring the sound speed and acoustic absorption [22]. His research points out a promising research direction for AT. Both sound absorption and sound speed need to be measured simultaneously in his reconstruction method. However, the measurement error of acoustic absorption is relatively large, making it less suitable as a measurement parameter for gases in complex environments [23]. The key research direction for the practical application of AT is to complete the imaging of gas concentration fields and temperature fields using only the speed of sound.

In excitable (diatomic or polyatomic molecule) gases, the relaxation process that occurs due to the energy exchange of the gases molecular vibrational modes causes the speed of sound to become dispersive [24,25,26]. This paper utilizes this characteristic to develop a reliable method for simultaneously reconstructing the temperature and concentration distributions of gas mixtures. The main contributions of this study are as follows:

1. The proposed method, based solely on the measurement of the speed of sound, can simultaneously reconstruct both the gas concentration and temperature fields.
2. The feasibility and effectiveness of this method have been verified through numerical simulation and experimental research.

## 2. Theoretical Background

### 2.1. Acoustic Relaxation Property

**Figure 1:**
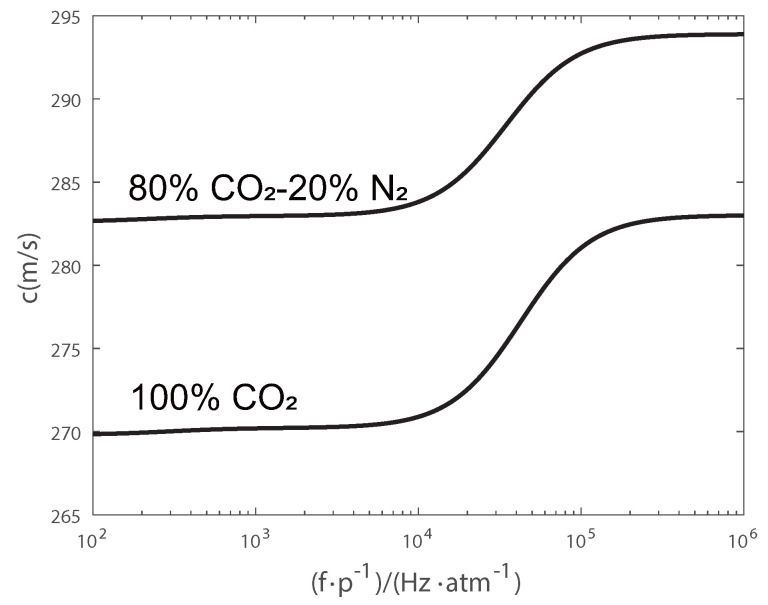
Frequency dependence of speed of sound for gases (T = 303 K).

The reconstruction of the gas concentration field and temperature field is based on the mathematical relationship of the speed of sound with gas concentration and temperature. The speed of sound can be expressed as follows [27]:

(1) $$c\left(\omega \right)=\sqrt{\frac{RT\gamma (\omega )}{M}},$$

where *M* and *T* are the molar mass and temperature of the gas; R represents the universal gas constant. $\gamma \left(\omega \right)=\left(C_{V}^{eff}\left(\omega \right)+R\right)/C_{V}^{eff}\left(\omega \right)$ is the heat capacity ratio, and ω=2πf is the acoustic angular frequency. $C_{V}^{eff}\left(\omega \right)$ is the effective isochoric specific heat. When sound waves propagate through excitable gases (polyatomic or diatomic), the delay between the activation and relaxation of internal degrees of freedom (primarily vibrational modes) leads to a loss of internal energy. During this process, energy is consumed in the form of heat, resulting in increased sound diffusion and absorption. According to acoustic relaxation theory, $C_{V}^{eff}\left(\omega \right)$ reflects the footprint of the molecular relaxation of gases. By adding the mole-fraction-weighted contributions from all gas molecules in the mixture, $C_{V}^{eff}\left(\omega \right)$ can be calculated as follows [28]:

(2) $$C_{V}^{eff}\left(\omega \right)=C_{V}^{\infty }+\sum_{k=1}^{W}\frac{a_{k}C_{k}^{int}}{1+i\omega \tau_{k}},C_{V}^{\infty }=\sum_{k=1}^{W}a_{k}C_{k}^{\infty },$$

where $a_{k}$ represents the mole fraction of molecule *k*, $\sum_{k=1}^{W}a_{k}=1$, and $C_{k}^{\infty }$ is the external specific heat of molecule *k*. $C_{k}^{\infty }$ depends on molecular symmetry, with a value of 5R/2 for a linear molecule and 3R for a nonlinear molecule. $\tau_{k}$ and $C_{k}^{int}$ are the relaxation time and internal specific heat of gas molecule *k*, respectively. Figure 1 shows the variation in c(ω) versus frequency, which results from acoustic relaxation.

### 2.2. Acoustic Tomography Model

As described above, the speed of sound is determined by the gas concentration and the ambient temperature. The entire speed of sound field in the area of interest affects the propagation time of the acoustic waves. AT reconstructs various physical parameters within the area of interest based on the propagation time of multiple acoustic propagation paths. Acoustic sensors are arranged around the area of interest to transmit and receive sound signals, obtaining the time of flight (TOF) for each propagation path.

**Figure 2:**
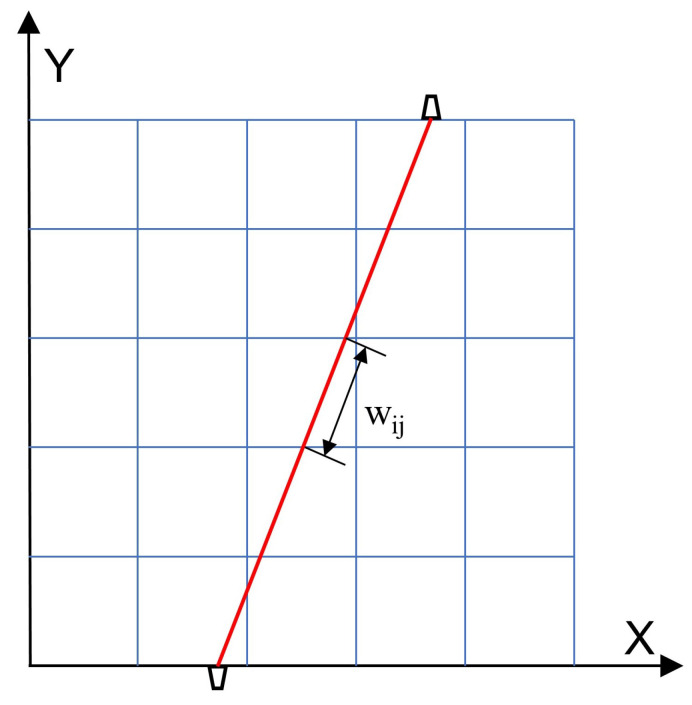
Discretization schematic of the AT problem.

The AT reconstruction process can be illustrated as follows: the area of interest is divided into several regions, as shown in Figure 2; $w_{ij}$ denotes the length of the *i*th path passing through the *j*th grid, and the coefficient matrix *W* is a collection of lengths under all paths and grids. The primary task of AT is to obtain the coefficient matrix *W* and establish a set of algebraic equations between the values of the speed of sound and the measured TOF.

Assuming there are *m* acoustic wave paths and *n* discrete regions within the area, we can derive the equation for the sound propagation time on the *i*th acoustic wave path as follows:

(3) $$t_{i}=\sum_{j=1}^{n}w_{ij}s_{j},i=1,2,\ldots ,m$$

where $s_{j}$ represents the reciprocal of the sound speed in *j*th grid. After a period of measurement, a system of equations for the *m* paths is obtained:

(4) $$\left\{\begin{array}{c} w_{11}s_{1}+w_{12}s_{2}+\ldots ,w_{1n}s_{n}=t_{1}\\ w_{21}s_{1}+w_{22}s_{2}+\ldots ,w_{2n}s_{n}=t_{2}\\ \vdots \\ w_{m1}s_{1}+w_{m2}s_{2}+\ldots ,w_{mn}s_{n}=t_{m} \end{array}\right.$$

The AT model can be simplified into the following matrix form:

(5) Wx=b

where the coefficient matrix $W\in R^{m\times n}$, $x={\left(s_{1},s_{2},\ldots ,s_{n}\right)}^{T}$ is a vector of the reciprocal of the speed of sound, and $b={\left(t_{1},t_{2},\ldots ,t_{m}\right)}^{T}$ is vector of the TOF data.

Considering the measurement error, Equation (5) can be modified by

(6) Wx=b+r

where *r* represents the measurement error or noise.

The major task of the inverse problem is to solve *x* from Equation (6). The least squares method (LSM) is currently a widely used reconstruction technique in AT. The advantages of the LSM include simple process, strong applicability, and stable results. Then, the initial reconstruction results can be further refined using interpolation methods, such as Gaussian process regression (GPR), to achieve refined results.

## 3. Simultaneous Reconstruction Method

### 3.1. Reconstruction for Binary Gas Mixture

**Figure 3:**
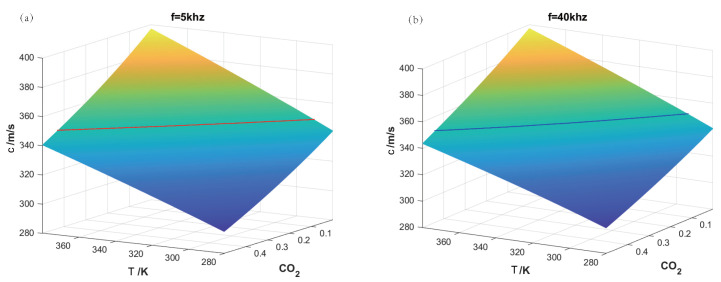
The relationship of the speed of sound with temperature and concentration. (**a**) f= 5 kHz; (**b**) f= 40 kHz.

The proposed method can accomplish the binary gas mixture concentration and temperature measurement solely by utilizing the dispersion of the speed of sound. Taking the $CO_{2}$–$N_{2}$ gas mixture as an example, Figure 3 displays the surfaces showing the variation in the speed of sound with $CO_{2}$ concentration and temperature. The two surfaces correspond to measurement frequencies of $f_{1}$ = 5 kHz and $f_{2}$ = 40 kHz, respectively. It can be observed that the characteristics of the two sound speed surfaces changes with $CO_{2}$ concentration and temperature are similar; that is, the speed of sound decreases as the $CO_{2}$ concentration increases and increases with rising temperature. At the same time, the surface for $f_{2}$ is slightly higher overall compared to the surface for $f_{1}$. It should be noted that although neither of the two surfaces is flat, there are no significant deformations or protrusions, which provides possibilities for the detection algorithm.

**Figure 4:**
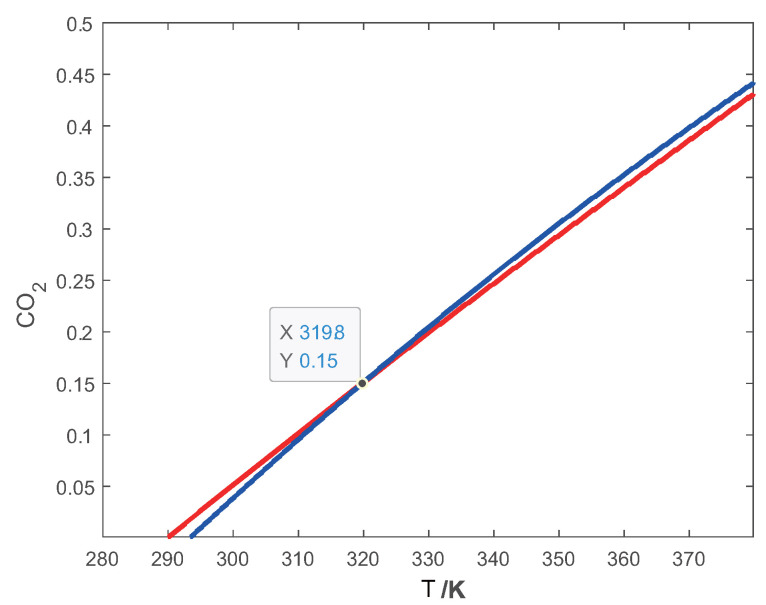
Projected curves for $CO_{2}$–$N_{2}$ gas mixture. The intersection of the two curves defines the concentration and temperature of the mixture.

For the test gas, 15%$CO_{2}$–85%$N_{2}$ at 319.8 K, the sound speeds at two frequency points were determined to be $c_{1}$ = 346.99 m/s and $c_{2}$ = 349.02 m/s, respectively. In Figure 3a, a horizontal slice of $c_{1}$ through the surface results in a red intersecting curve. The curve represents the concentration and temperature conditions of the gas mixture satisfying *c* = 346.99 m/s with *f* = 5 kHz. Similarly, in Figure 3b, an intersecting curve for $c_{2}$ = 349.02 m/s is created, resulting in a blue curve parallel to the base plane. Both curves have the same coordinate range for the base plane, allowing the projection of both curves onto the same base plane, as shown in Figure 4. The intersection point of the red and blue curves is the concentration of $CO_{2}$ at 15% and a temperature of 319.8 K. Therefore, this method is also named as the projection intersection method.

### 3.2. Reconstruction for Ternary Gas Mixture

In Section 3.1, the projection intersection method used the speed of sound at two frequencies to measure gas concentration and temperature. This subsection continues to apply this graphical algorithm to measure three physical parameters in a ternary gas mixture: temperature and the concentrations of two gas components. By utilizing the variation in the speed of sound with temperature and concentration, the corresponding sound speed for the test gas are measured and the parameter models that satisfy the conditions are identified. The obtained parameter models are then placed within the same coordinate plane. The coordinates of the intersection point obtained in this manner represent the temperature and concentration parameters of the test gas.

Figure 3 presents a three-dimensional model of the speed of sound in relation to concentration and temperature in a binary gas mixture. When extended to a ternary gas mixture, an additional concentration parameter is introduced, and the function of the speed of sound with respect to the three physical parameters becomes a four-dimensional model. This is a three-dimensional body with varying grayscale values, which is not convenient for graphical representation. As indicated in Section 3.1, the focus of the graphical algorithm is to build the parameter model corresponding to the measured values.

**Figure 5:**
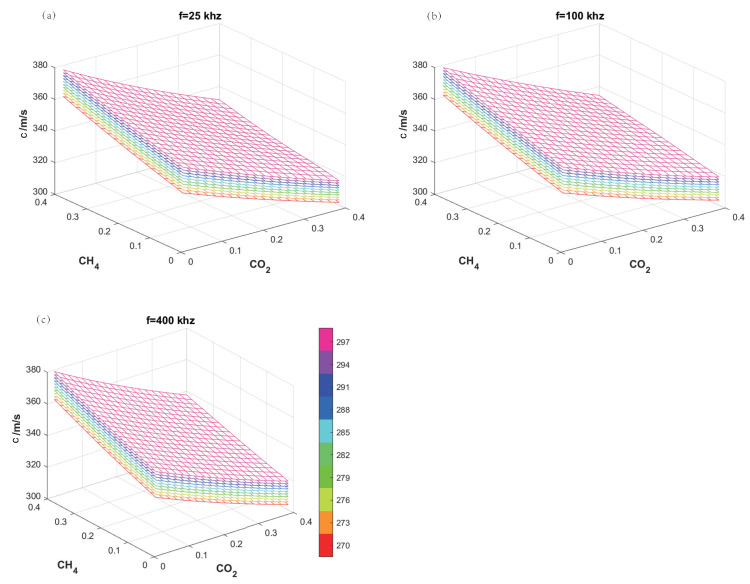
The relationship of the speed of sound with temperature and concentration. (**a**) f= 5 kHz; (**b**) f= 100 kHz; (**c**) f= 400 kHz.

Taking the $CO_{2}$–$CH_{4}$–$N_{2}$ gas mixture as an example, Figure 5a illustrates the relationship between the speed of sound at 25 kHz and the concentrations of $CO_{2}$ and $CH_{4}$, as well as the temperature. The x-axis and y-axis represent the concentrations of $CO_{2}$ and $CH_{4}$, respectively, while the z-axis represents the speed of sound. Temperature is represented by color, with a total of 10 temperature surfaces, the lowest being at 270 K and the highest at 297 K, with an interval of 3 K between adjacent surfaces. This visual representation of the physical parameter of temperature provides convenience for subsequent measurement methods. It can be observed that as the temperature rises, the speed of sound increases. The surfaces at the 10 different temperatures do not overlap, with no significant deformations or protrusions. For the surfaces at a single temperature, the speed of sound decreases with increasing $CO_{2}$ concentration and increases with increasing $CH_{4}$ concentration. Additionally, Figure 5a indicates that once the speed of sound is determined, the gas concentrations and temperature that satisfy the speed of sound are not fixed. Similar to Figure 5a, Figure 5b,c present the relationship of the speed of sound with temperature and concentration at 100 kHz and 400 kHz, respectively. The characteristics in the three figures are similar. At the same temperature and concentration, the higher the frequency, the higher the speed of sound.

**Figure 6:**
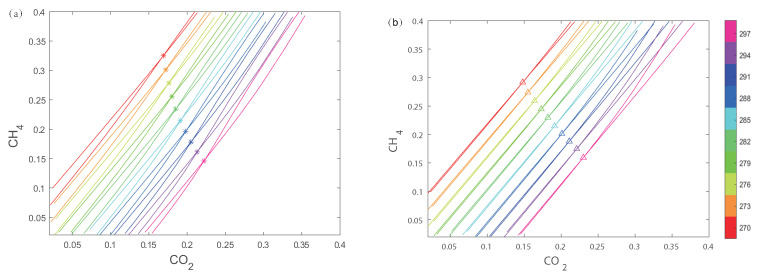
The intersection curves of the speed of sound are projected onto the base plane. (**a**) Curves $l_{1}$ and $l_{2}$; (**b**) curves $l_{2}$ and $l_{3}$.

For a test gas at 285 K with a composition of 19%$CO_{2}$–21%$CH_{4}$–60%$N_{2}$, speeds of sound at different frequencies are obtained as follows: $c_{1}$ = 337.33 m/s at 25 kHz, $c_{2}$ = 339.03 m/s at 100 kHz, and $c_{3}$ = 339.25 m/s at 400 kHz. In Figure 5a, an isosurface for $c_{1}$ is created, which intersects with the surfaces at ten different temperatures, yielding a set of non-intersecting curves $l_{1}$, each with a color corresponding to its respective temperature surface. The same procedure is applied in Figure 5b to create an isosurface for $c_{2}$, resulting in a set of curves $l_{2}$, and in Figure 5c for $c_{3}$, yielding another set of curves $l_{3}$. As shown in Figure 6a, both sets of curves $l_{1}$ and $l_{2}$ are projected onto the same plane, with the intersection points of curves of the same color marked with asterisks, indicating the conditions satisfied by the sound speed at both frequencies. Similarly, the two sets of curves $l_{2}$ and $l_{3}$ are projected onto the same plane, and the intersection points of curves with the same color are marked with upper triangles, as illustrated in Figure 6b.

**Figure 7:**
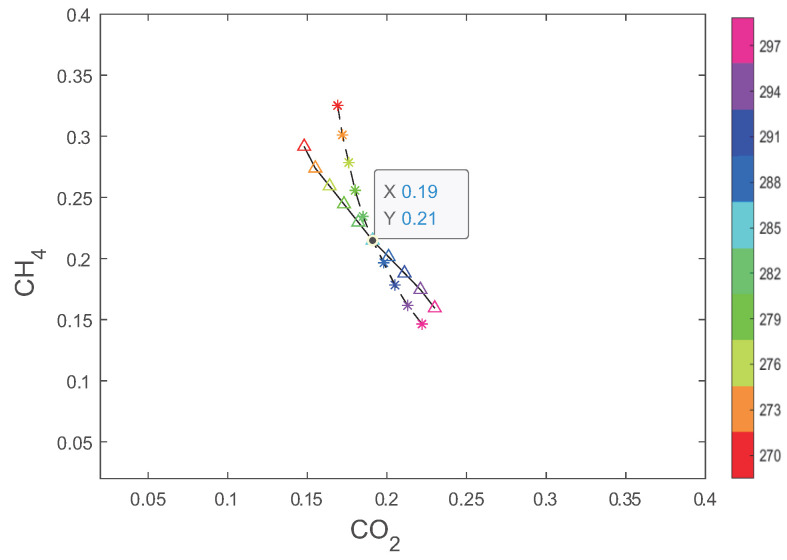
Projected curves for $CO_{2}$–$CH_{4}$–$N_{2}$ gas mixture.

To clearly present the intersection points, in Figure 7 the three sets of intersection lines are removed, leaving only the asterisks and upper triangles, which are connected with dashed and solid lines, respectively. The intersection point of the dashed and solid lines indicates the conditions satisfied by the speed of sound at all three frequencies. The horizontal and vertical coordinates represent the concentrations of $CO_{2}$ and $CH_{4}$, respectively, and the color indicates the temperature. It can be seen that $CO_{2}$ concentration is 19%, $CH_{4}$ concentration is 21%, and the temperature is 285 K. The algorithm has determined the concentrations and temperature of the ternary gas mixture. Moreover, in practical applications, interpolation methods can be used to solve for temperature values that are not within the preset range.

## 4. Numerical Simulations and Discussion

### 4.1. Reconstruction Procedure and Details

The method for reconstructing temperature and concentration fields based on the dispersion of the speed of sound is described as follows:

1. Divide the test area into *n* discrete small regions;
2. Arrange an array of acoustic sensors with multiple frequencies within the area of interest, forming *m* acoustic paths for each frequency;
3. Measure the propagation time of each path to obtain a vector of sound propagation times for each frequency;
4. Calculate the coefficient matrix *W*;
5. Use the LSM to solve Equation (5), obtaining a vector of the reciprocal of the speed of sound for each frequency, thus obtaining the multi-frequency speeds of sound in each grid;
6. Employ the GPR interpolation method to obtain the reconstructed speed of sound field with a refined grid;
7. Utilize the projection intersection method to determine the physical parameters in the refined grids, thereby obtaining the results for the concentration and temperature fields.

To evaluate the reconstruction quality of the gas concentration and temperature fields, we employ the following three indicators to analyze the results: the maximum relative error $E_{max}$, the mean relative error $E_{mean}$, and the root mean square error $E_{rms}$. Their calculation formulas are as follows:

(7) $$E_{max}=max\left|\frac{C\left(i\right)-C^{\prime }\left(i\right)}{C(i)}\right|\times 100\%$$

(8) $$E_{mean}=\frac{\sum_{i=1}^{n}\left|\frac{C\left(i\right)-C^{\prime }\left(i\right)}{C(i)}\right|}{n}\times 100\%$$

(9) $$E_{rms}=\frac{\sqrt{\frac{1}{n}\sum_{i=1}^{n}{\left(C\left(i\right)-C^{\prime }\left(i\right)\right)}^{2}}}{C_{ave}}\times 100\%$$

where C(i) represents the true value of the physical parameter (concentration or temperature) at the *i*-th grid point, $C^{\prime }\left(i\right)$ represents the measured value of the physical parameter at the *i*-th grid point, and $C_{ave}$ represents the average value in the distribution model.

**Figure 8:**
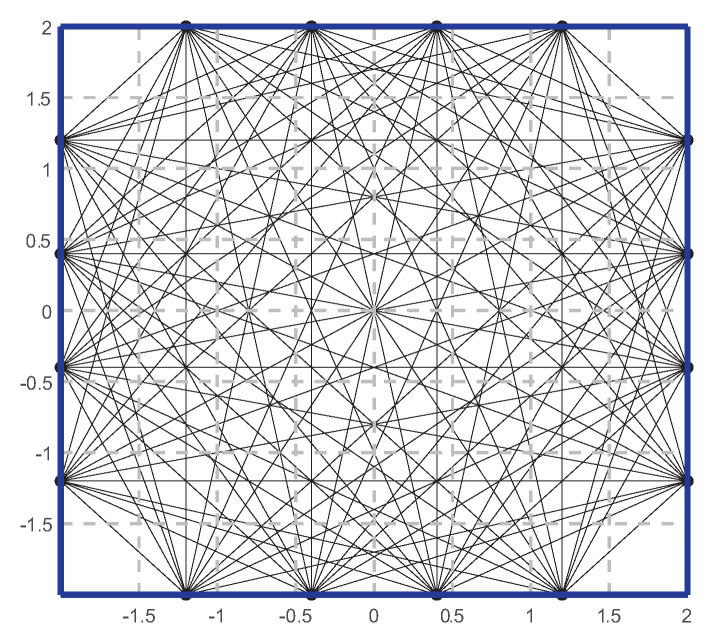
Arrangement of the acoustic transceivers.

In this section, numerical simulation experiments are utilized to evaluate the simultaneous reconstruction method for temperature and concentration fields, and the calculation process is performed using the MATLAB software. As shown in Figure 8, the measurement area in the experiment is a square of 4 m × 4 m, with the center of the area as the origin. There are 16 acoustic transceivers evenly distributed around the periphery, each capable of transmitting and receiving sound waves at multiple frequencies. When one of the transceivers emits a sound wave, the transducers on the other three sides act as receivers to capture the flight time, resulting in a total of 96 effective propagation paths. The area of interest is divided into 8×8=64 coarse grids and 81×81=6561 refined grids.

In actual acoustic measurements, TOF data are subject to the influence of equipment precision and environmental interference. In numerical simulations, Gaussian white noise with a mean of 0 and a variance of $1\times 10^{-4}$ is introduced into the TOF data.

### 4.2. AT for Binary Gas Mixture

In industrial production, the concentration and temperature fields are continuous. To verify the universality of the method, for the $CO_{2}$–$N_{2}$ gas mixture, we select three typical distributions for reconstruction experiments. The first is a single-peak symmetric distribution, where both the temperature and concentration fields have only one peak value. The second is a double-peak symmetric distribution, composed of two regions with the same peak value. The third is a double-peak skewed distribution, consisting of two regions with different peak values. The mathematical formulas for the three distributions are as follows:

The single-peak symmetry distribution:

(10) $$\begin{array}{c} C\left(x,y\right)=0.1+0.2e^{-3(x^{2}+y^{2})} \end{array}$$

(11) $$\begin{array}{c} T\left(x,y\right)=300+35e^{-3(x^{2}+y^{2})} \end{array}$$

The double-peak symmetric distribution:

(12) $$\begin{array}{c} C\left(x,y\right)=0.2+0.2e^{-3({\left(x-0.75\right)}^{2}+{\left(y-0.75\right)}^{2})}+0.2e^{-3({\left(x+0.75\right)}^{2}+{\left(y+0.75\right)}^{2})} \end{array}$$

(13) $$\begin{array}{c} T\left(x,y\right)=300+30e^{-3({\left(x-0.75\right)}^{2}+{\left(y-0.75\right)}^{2})}+30e^{-3({\left(x+0.75\right)}^{2}+{\left(y+0.75\right)}^{2})} \end{array}$$

The double-peak skewed distribution:

(14) $$\begin{array}{c} C\left(x,y\right)=0.2+0.2e^{-3({\left(x-0.75\right)}^{2}+{\left(y-0.75\right)}^{2})}+0.15e^{-3({\left(x+0.75\right)}^{2}+{\left(y+0.75\right)}^{2})} \end{array}$$

(15) $$\begin{array}{c} T\left(x,y\right)=300+30e^{-3({\left(x-0.75\right)}^{2}+{\left(y+0.75\right)}^{2})}+25e^{-3({\left(x+0.75\right)}^{2}+{\left(y-0.75\right)}^{2})} \end{array}$$

where C(x,y) represents the distribution of the $CO_{2}$ concentration, and T(x,y) represents the distribution of temperature.

**Figure 9:**
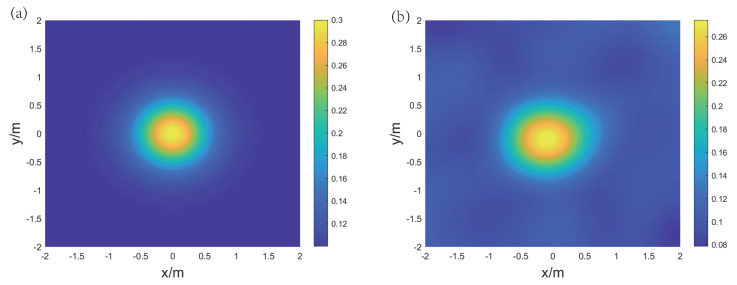

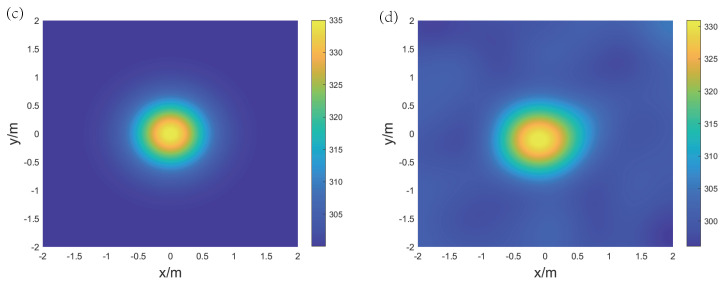
The original and reconstructed state fields under the single-peak symmetric distribution. (**a**) Original concentration field, (**b**) reconstructed concentration field, (**c**) original temperature field, and (**d**) reconstructed temperature field.

**Figure 10:**
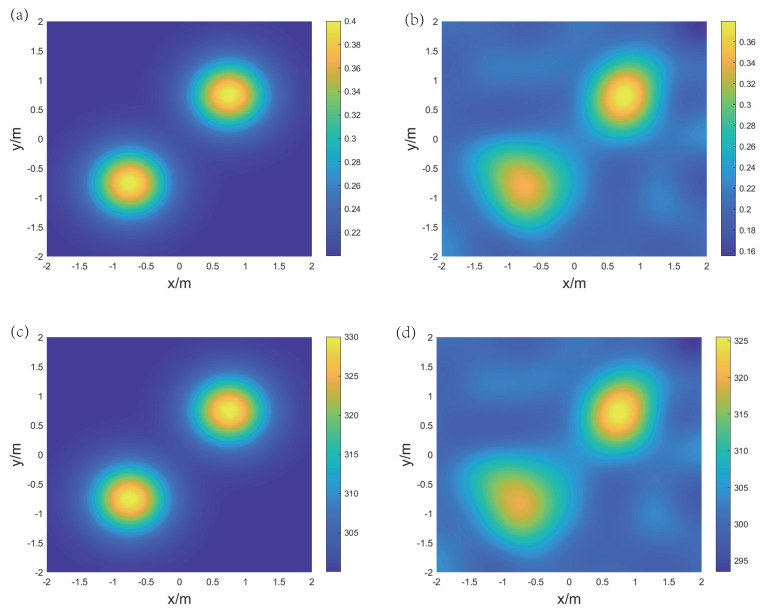
The original and reconstructed state fields under the double-peak symmetric distribution. (**a**) Original concentration field, (**b**) reconstructed concentration field, (**c**) original temperature field, and (**d**) reconstructed temperature field.

**Figure 11:**
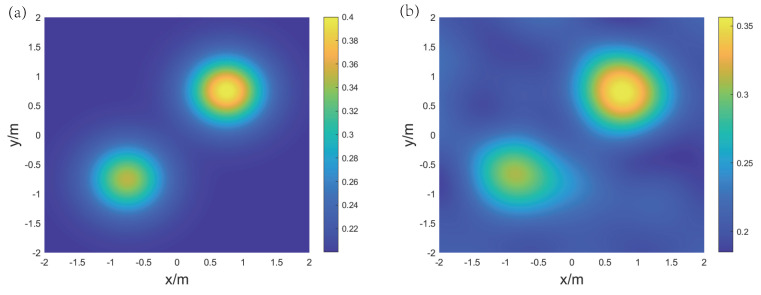

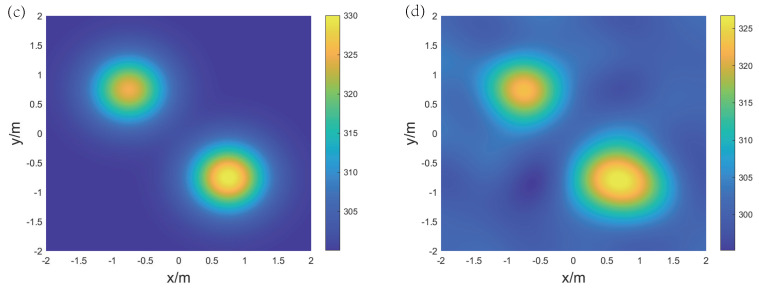
The original and reconstructed state fields under the double-peak skewed distribution. (**a**) Original concentration field, (**b**) reconstructed concentration field, (**c**) original temperature field, and (**d**) reconstructed temperature field.

For the reconstruction of temperature and concentration fields for the binary gas mixture, two frequencies are selected: $f_{1}$ = 5 kHz and $f_{2}$ = 40 kHz. Figure 9, Figure 10, and Figure 11, respectively, display the original and reconstructed fields of the gas under the three distributions, and Table 1 lists the $E_{max}$, $E_{mean}$, and $E_{rms}$ for the aforementioned reconstruction results.

The results indicate that in the single-peak symmetric distribution, the reconstructed concentration and temperature fields are consistent with the original distribution. The peak positions for temperature and concentration are identical. The high concentration of $CO_{2}$ leads to a decrease in the speed of sound, while high temperature causes an increase in the speed of sound. However, the proposed method can effectively decouple these two effects. The mean relative errors $E_{mean}\left(CO_{2}\right)$ and $E_{mean}\left(T\right)$ are 6.99% and 0.53%, respectively, indicating that the overall quality of the reconstruction is good. Meanwhile, the maximum relative error $E_{max}\left(CO_{2}\right)$ is 26.77%, which suggests that the reconstruction effect in some points is unstable. This is because the initial grid division is not refined enough, leading to errors in the process of the speed of sound inversion. Additionally, the peak points are located at the intersections of the grids, further exacerbating the negative impact of grid division.

For the double-peak symmetric distribution, the peak positions of the reconstructed concentration and temperature fields are consistent with the original distribution, but the transition from the peaks to the low points is more gradual. The root mean square errors $E_{rms}\left(CO_{2}\right)$ and $E_{rms}\left(T\right)$ are 3.7% and 0.6%, respectively, indicating that the reconstruction errors of concentration and temperature over the area of interest are relatively low. Notably, in the upper right corner region of both reconstruction results there are values that are significantly lower than those of the original distribution. This occurs because the LSM loses edge information during the reconstruction of the speed of sound field, and GPR is influenced by nearby peak points when fitting the distribution.

For the double-peak skewed distribution, the reconstructed concentration and temperature fields not only accurately restore the positions of the two peak points but also effectively depict the relative magnitudes of these peaks. Additionally, the mean relative errors $E_{mean}\left(CO_{2}\right)$ and $E_{mean}\left(T\right)$ are 4.55% and 0.6%, respectively, indicating that the reconstruction quality is good in various regions. In summary, the proposed method can achieve excellent results for concentration and temperature fields under different working conditions.

### 4.3. AT for Ternary Gas Mixture

For the $CO_{2}$–$CH_{4}$–$N_{2}$ gas mixture, we select two typical distributions for reconstruction experiments: single-peak symmetric and double-peak symmetric. The mathematical formulas for the two distributions are as follows:

The single-peak symmetry:

(16) $$\begin{array}{c} C_{1}\left(x,y\right)=0.2+0.2e^{-3(x^{2}+y^{2})} \end{array}$$

(17) $$\begin{array}{c} C_{2}\left(x,y\right)=0.2+0.2e^{-3(x^{2}+y^{2})} \end{array}$$

(18) $$\begin{array}{c} T\left(x,y\right)=275+10e^{-3(x^{2}+y^{2})} \end{array}$$

The double-peak symmetry:

(19) $$\begin{array}{c} C_{1}\left(x,y\right)=0.2+0.1e^{-3({\left(x-0.75\right)}^{2}+{\left(y-0.75\right)}^{2})}+0.1e^{-3({\left(x+0.75\right)}^{2}+{\left(y+0.75\right)}^{2})} \end{array}$$

(20) $$\begin{array}{c} C_{2}\left(x,y\right)=0.2+0.1e^{-3({\left(x-0.75\right)}^{2}+{\left(y-0.75\right)}^{2})}+0.1e^{-3({\left(x+0.75\right)}^{2}+{\left(y+0.75\right)}^{2})} \end{array}$$

(21) $$\begin{array}{c} T\left(x,y\right)=280+10e^{-3({\left(x-0.75\right)}^{2}+{\left(y-0.75\right)}^{2})}+10e^{-3({\left(x+0.75\right)}^{2}+{\left(y+0.75\right)}^{2})} \end{array}$$

where $C_{1}\left(x,y\right)$, $C_{2}\left(x,y\right)$ represent distributions of $CO_{2}$ and $CH_{4}$ concentration, T(x,y) represents the distribution of temperature.

**Figure 12:**
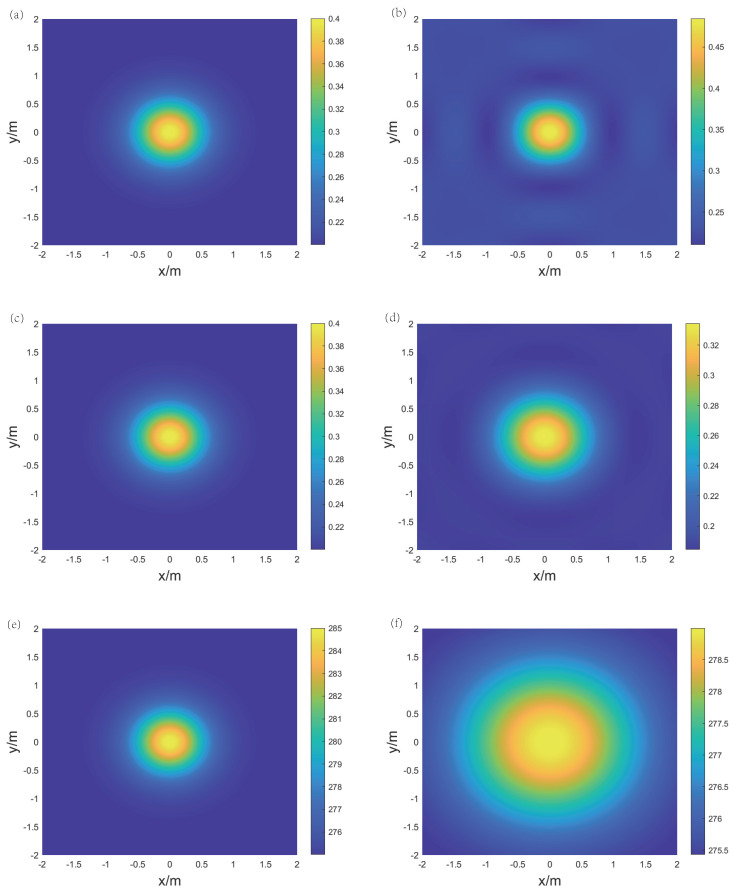
The original and reconstructed fields under the single-peak symmetric distribution. (**a**) Original $CO_{2}$ concentration field, (**b**) reconstructed $CO_{2}$ concentration field, (**c**) original $CH_{4}$ concentration field, (**d**) reconstructed $CH_{4}$ concentration field, (**e**) original temperature field, and (**f**) reconstructed temperature field.

**Figure 13:**
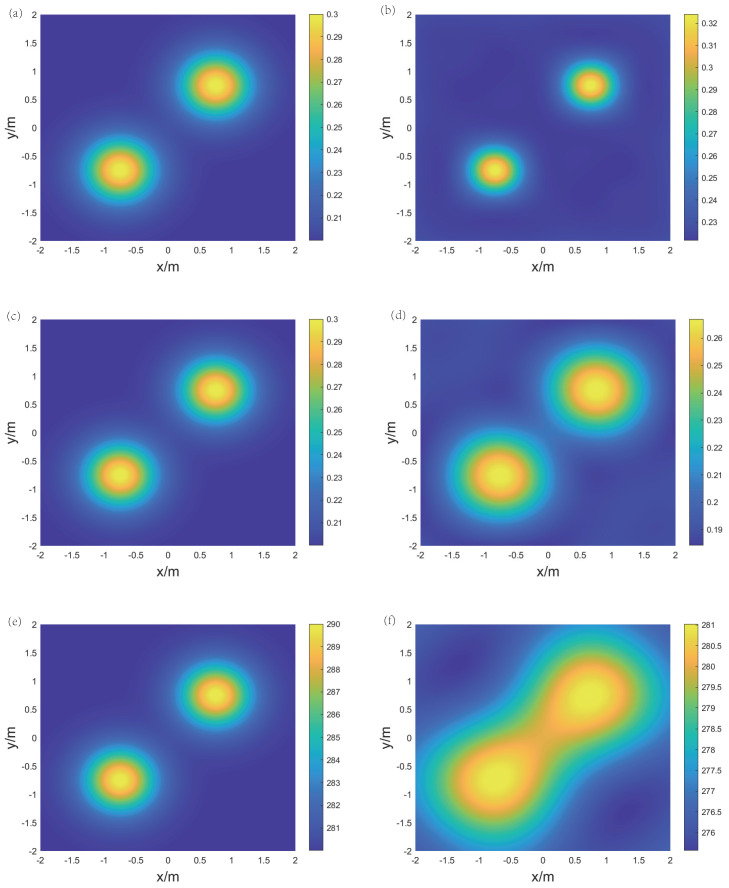
The original and reconstructed fields under the double-peak symmetric distribution. (**a**) Original $CO_{2}$ concentration field, (**b**) reconstructed $CO_{2}$ concentration field, (**c**) original $CH_{4}$ concentration field, (**d**) reconstructed $CH_{4}$ concentration field, (**e**) original temperature field, and (**f**) reconstructed temperature field.

For the reconstruction of temperature and concentration fields for the ternary gas mixture, three frequencies are selected: $f_{1}$ = 25 kHz, $f_{2}$ = 100 kHz, and $f_{3}$ = 400 kHz. Figure 12 and Figure 13, respectively, display the original and reconstructed fields of the gas under the two distributions, and Table 2 lists the $E_{max}$, $E_{mean}$, and $E_{rms}$ for the aforementioned reconstruction results.

The results show that for the single-peak symmetric distribution, the shape of the reconstructed $CO_{2}$ concentration field is consistent with the original distribution, and the peak positions of the $CH_{4}$ concentration field and temperature field are consistent with the original distribution. However, the transition from the peak values to the low points is more gradual. The maximum relative error $E_{max}\left(CO_{2}\right)$ is 20.25%, mainly due to the concentration at the central peak point being overestimated. The mean relative error $E_{mean}\left(CH_{4}\right)$ and the root mean square error $E_{rms}\left(CH_{4}\right)$ are 5.61% and 5.77%, respectively, indicating that the reconstruction result of the $CH_{4}$ concentration field is overall good.

For the double-peak symmetric distribution, the reconstructed temperature field exhibits a connection between the two peak areas due to the expanded range of the Gaussian distribution. The shapes of both concentration fields remain consistent with the shape of the original distribution. In terms of reconstruction errors, $E_{mean}\left(CO_{2}\right)$ and $E_{rms}\left(CO_{2}\right)$ are 4.43% and 5.18%, respectively, while $E_{mean}\left(CH_{4}\right)$ and $E_{rms}\left(CH_{4}\right)$ are 8.39% and 8.92%, respectively. This indicates that the overall reconstruction result of the $CO_{2}$ concentration field is better than that of the $CH_{4}$ concentration field. In summary, in the reconstruction of temperature and concentration fields for a ternary gas mixture, measurement errors have a significant impact on local reconstruction results, but it is still possible to obtain the distribution trends in concentration and temperature within the area.

## 5. Experimental Study

To validate the effectiveness of the proposed method based on the speed of sound dispersion, an AT system for two-dimensional concentration and temperature fields was designed. An electric furnace and gas cylinders were used within the area of interest to simulate changes in the temperature and gas concentration. Acoustic measurements were taken to obtain the physical fields, and the experimental outcomes were analyzed.

### 5.1. Apparatus

The AT system consists of two subsystems, namely, the ultrasonic system and the audible sound system. These two subsystems are responsible for measuring the speed of sound field in the ultrasonic and audible frequency bands, respectively, to obtain the speed of sound dispersion data on the grid points within the area of interest, and ultimately calculate the gas concentration and temperature.

**Figure 14:**
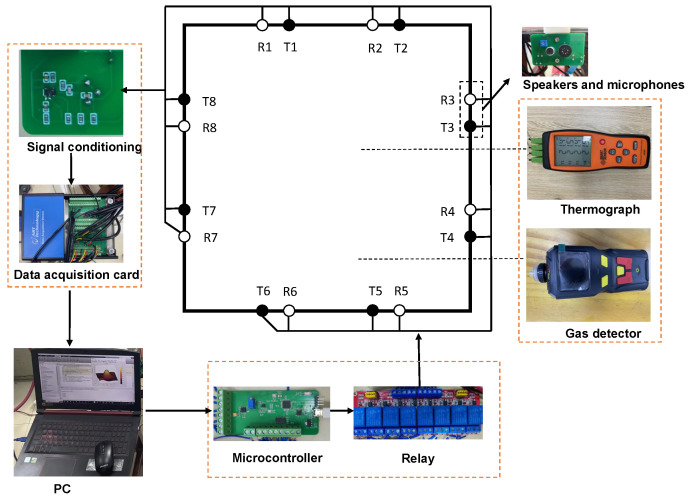
Schematic of audible sound system. The system includes transmitting circuit, receiving circuit, and standard measuring instrument.

The block diagram of the audible sound system is shown in Figure 14; it mainly includes speakers, microphones, the data acquisition card, a relay, a signal controller, and a signal conditioner. The speaker selected has a frequency response range of 500–20 kHz and a peak power output of 1.2 W. The capacitive microphone has a frequency response range of 50–20 kHz and a signal-to-noise ratio of 58 dB. A pair of speaker and microphone serve as a single transceiver unit, fixed on the signal conditioning circuit board. A square aluminum alloy frame, measuring 1 m×1 m, has two transceiver units installed on each side. The computer controls the signal generation chip (AD9833) to produce a sine wave with an amplitude of 0.6 V through a microcontroller (STM32F103), and the amplitude is increased to 2.5 V after passing through the power amplification circuit (LM386). The sound from the speaker is controlled by an 8-channel relay, allowing the eight speakers to emit sound wave signals sequentially. After passing through the area of interest, the sound signals are picked up by all other microphones. A preamplification circuit composed of an audio amplification chip (OPA1671) amplifies the received signals, which are then converted into digital signals by the data acquisition card and sent to the computer. The data acquisition card, produced by Art Technology, can synchronously collect 32 channel signals, with a maximum sampling rate of 500 kS/s.

The ultrasonic system is similar to the audible sound measurement system. The ultrasonic system utilizes 16 transceiver ultrasonic sensors with a frequency of 25 kHz. Eight sensors are used as transmitters and eight as receivers. The ultrasonic sensors are installed on the same plane as the audible sound sensors, but their positions are staggered. Unlike the audible sound sensors, ultrasonic sensors require a higher power drive. Therefore, the signal is first amplified to 10 V by a power amplifier circuit (TPA3255) before being sent to the 8-channel relay circuit to select the corresponding sensors. For convenience in controlling the emission and collection of signals, the ultrasonic system and the audible sound system share the same computer and acquisition card.

**Figure 15:**
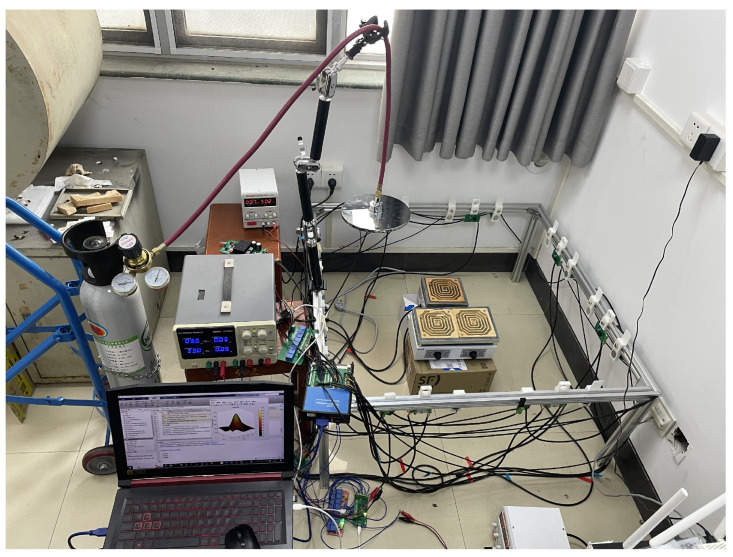
Picture of the AT system. This figure shows the scene of the experiment.

Figure 15 shows the picture of the AT system. The workflow of the system is as follows: The computer first completes the measurement of the audible sound system. Computer controls the audible sound control circuit to generate sine wave signals at the selected frequency. After passing through amplification circuits and enabling circuits, the speakers emit signals in turn, while the microphones receive signals during this process. The signals are then amplified, filtered, and collected by the data acquisition card. The ultrasonic system measurement is carried out after the completion of the audible sound system measurement, and the total time used by both systems constitutes a complete measurement period.

To verify the results, measurements of concentration and temperature in the area of interest were conducted. The concentration of $CO_{2}$ was measured using a portable gas detector (JES-MS400HS-CO2), with a measurement range of 0–100% VOL and a resolution of 0.01% VOL. Temperature measurement was carried out using a thermocouple contact thermometer (Smart-AS887), which supports four-channel synchronous temperature measurement with a total range of −50 to 300 °C.

### 5.2. Experimental Results

The imaging area is 1 m×1 m, with a total of 24 effective acoustic paths, divided into 3×3 coarse grids and 16×16 fine grids. Below the imaging area, three electric heaters, each with a power of 1 kW, are arranged to serve as a heat source, with the imaging plane 15 cm above the top of the heaters. Simultaneously, a gas cylinder providing 99.99% $CO_{2}$ is used as the gas source above the imaging area, with the cylinder’s outlet connected to a showerhead to achieve a stable airflow, serving to increase the area of gas distribution. The gas flow rate is controlled by a pressure reducing valve, maintaining stable pressure during the experiment. In the experiment, thermocouples are used to measure the temperature at four points within the imaging area, and a gas detector is used to measure the $CO_{2}$ concentration at the gas source location within the imaging plane. During TOF data measurements, the length of the signal sequence for each frame is 1 ms. The interval between the two adjacent transmissions is 30 milliseconds to avoid overlapping of the received signals.

**Figure 16:**
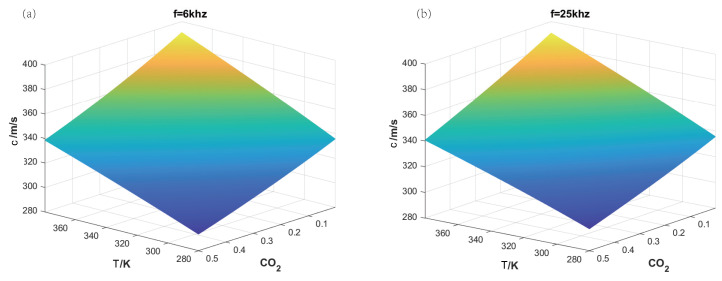
The relationship of the speed of sound with temperature and concentration for carbon dioxide and air gas mixture. (**a**) Frequency of 6 kHz; (**b**) frequency of 25 kHz.

The projection intersection method for the concentration and temperature fields of gas mixture is the same as in Section 3.1. Air contains multiple components, but a reduction method can be used, i.e., considering it as a single type of gas. Therefore, air can be regarded as a single-component gas with fixed concentration ratios of nitrogen and oxygen at 78:22. The concentration variation ranges for the three gases within the test area are defined as $CO_{2}$: $a_{1}=0-100\%$; $N_{2}$: $a_{2}=0.78\left(1-a_{1}\right)$; and $O_{2}$: $a_{3}=0.22\left(1-a_{1}\right)$. For the two chosen acoustic frequency points, $f_{1}$ = 6 kHz and $f_{2}$ = 25 kHz, Figure 16 presents a three-dimensional model of the speed of sound and concentration and temperature of the test gas. It can be seen that the three-dimensional models at the two frequencies are similar to those in Section 3.1 and are applicable to the binary gas sensing method.

**Figure 17:**
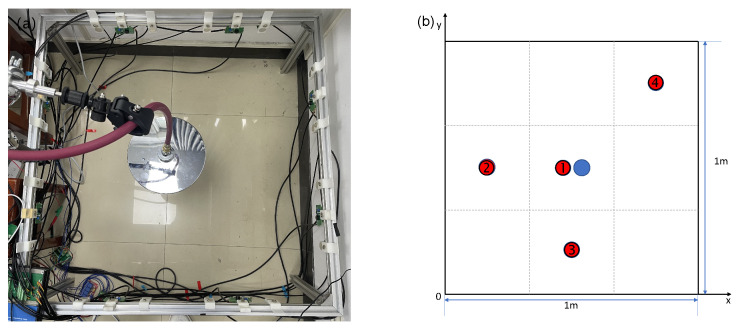
(**a**) Layout photo of Experiment 1. (**b**) Measurement point location diagram of Experiment 1. Red: thermocouple measurement points nos. 1–4; blue: $CO_{2}$ concentration measurement points.

**Figure 18:**
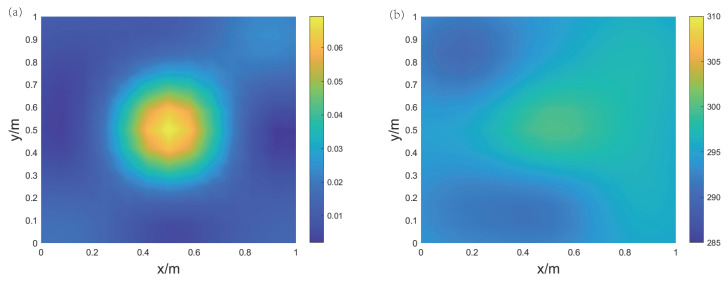
Reconstruction results of Experiment 1 for air and carbon dioxide gas mixture. (**a**) $CO_{2}$ concentration field; (**b**) temperature field.

Experiment 1 is a single-gas-source experiment, without the electric heater. As shown in Figure 17a, the gas source is aimed at the center of the region. The measurement points are as shown in Figure 17b, and the measurement values of the five points along with the corresponding reconstruction results are listed in Table 3. Figure 18 displays the imaging results of the two-dimensional $CO_{2}$ concentration and temperature distribution. It can be observed from Figure 18 that the peak in the concentration field is in the middle, consistent with the expected results. The high concentration of $CO_{2}$ is quickly diluted by the surrounding air in the open environment, with a peak value of only 8%, which is close to the measurement results. Since there is no heat source, the overall temperature of the target area remains stable around 293 K. The reconstructed temperature field values range from 290 to 300 K, which is close to the measurement results, with the errors are within 7 K at the four measurement points. It can be seen that in the case of only having one gas source the overall trend is consistent with expectations.

**Figure 19:**
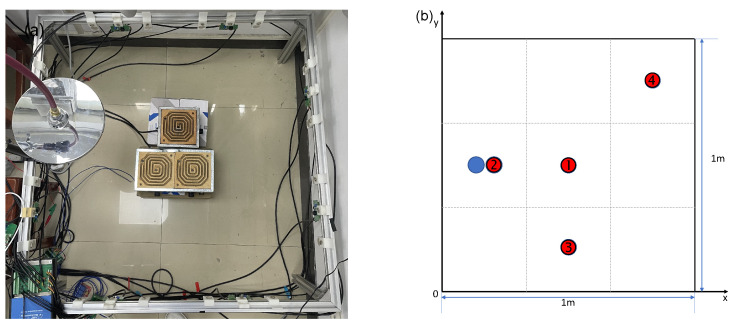
(**a**) Layout photo of Experiment 2. (**b**) Measurement point location diagram of Experiment 2. Red: thermocouple measurement points nos. 1–4; blue: $CO_{2}$ concentration measurement points.

**Figure 20:**
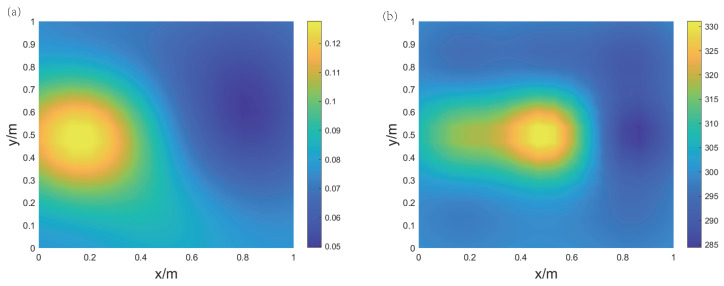
Reconstruction results of Experiment 2 for air and carbon dioxide gas mixture. (**a**) $CO_{2}$ concentration field; (**b**) temperature field.

Experiment 2 utilizes both a gas source and a heat source, with the electric heater positioned at the center of the imaging area, and the gas source aimed at the position to the left of the heater, as shown in Figure 19a. The measurement points are as depicted in Figure 19b. Figure 20 presents the imaging results of the $CO_{2}$ concentration and temperature distribution. It is evident that the peak in the temperature is in the center of the region, while the peak in the concentration is on the left side. The concentration and temperature peaks in the imaging results are in agreement with the actual conditions, demonstrating the practicality of this method in reconstructing the concentration and temperature distribution of gas mixtures. The measurement values of the five measurement points and the corresponding reconstruction results are listed in Table 4. The results from the table indicate that compared to the measurements from the thermocouples, the relative errors in temperature at four locations are within 5%. The error at the position of the heat source (temperature measurement point 1) is larger, while the error at the position furthest from the heat source (temperature measurement point 4) is smaller. The standard concentration measurement result is close to the reconstruction results. The results of Experiment 2 demonstrate that the acoustic imaging system constructed can effectively achieve imaging of the concentration field and temperature field for binary gas mixtures.

**Figure 21:**
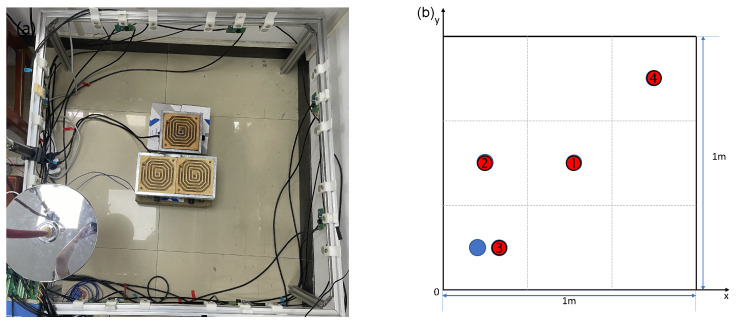
(**a**) Layout photo of Experiment 3. (**b**) Measurement point location diagram of Experiment 3. Red: thermocouple measurement points nos. 1–4; blue: $CO_{2}$ concentration measurement points.

**Figure 22:**
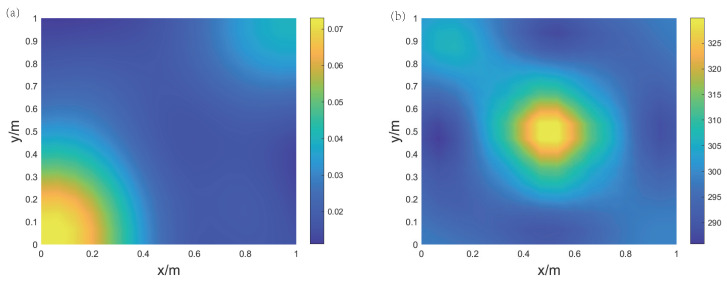
Reconstruction results of Experiment 3 for air and carbon dioxide gas mixture. (**a**) $CO_{2}$ concentration field; (**b**) temperature field.

Experiment 3 places the electric heater at the center of the imaging area, with the gas source aimed at the lower left corner of the heater, as shown in Figure 21a. The measurement points are as indicated in Figure 21b, and the measurement values of the five points along with the corresponding reconstruction results are listed in Table 5. Figure 22 displays the imaging results of the two-dimensional $CO_{2}$ concentration and temperature distribution. From Figure 22, it can be observed that the peak in the concentration field is in the lower left corner, consistent with the expected results. The peak concentration is 6.3%, which is close to the measurement results, with an error of 1.1%. Temperature measurement point 1 corresponds to the heating position of the electric heater, with an error of 5 K compared to the reconstruction results; the temperatures at the three edge positions are 30 K lower than the center temperature, and the error is within 8 K when compared to the reconstruction results.

Based on the results of the three experiments mentioned above, it can be concluded that the constructed imaging system is capable of effectively imaging the concentration and temperature of gases under different conditions. However, there is still significant room for improvement in the measurement accuracy of the experimental system. The main sources of error are as follows:

(1) Noise interference. The start point of the signal reception in TOF (time-of-flight) measurement is susceptible to environmental noise, which can affect the accuracy of the measurements.

(2) Limited number of sensors. The simultaneous measurement of two frequencies leads to an insufficient number of sensors for a single frequency, making it difficult to achieve fine imaging of the target area. Increasing the number and distribution of sensors could enhance the resolution and accuracy of the imaging results.

(3) Instability of concentration. In open spaces, it is challenging to maintain absolute stability of airflow, which can lead to variations in gas concentration. Dynamic gas imaging algorithms are needed to mitigate the impact of concentration changes and to provide more stable and accurate measurements.

## 6. Conclusions

This paper begins by introducing the basic principles of AT, including the reconstruction method LSM and interpolation method GPR. Utilizing the dependence of the speed of sound at two frequency points on the concentration and temperature of a binary gas mixture, three-dimensional models of the speed of sound with gas concentration and temperature are established. The projection intersection method is used for calculating the concentration and temperature by measuring the speed of sound dispersion. This graphical algorithm is extended to the detection of the concentration and temperature of a ternary gas mixture. The function of the speed of sound at three frequency points and the concentration and temperature in a ternary gas mixture belongs to a four-dimensional model, which is a three-dimensional body with grayscale changes that is not convenient for graphical presentation. The variable temperature is represented using gradient colors to establish a three-dimensional model of the speed of sound with two concentration variables and one temperature variable. Then, by calculating the common intersection points of the speed of sound at three frequency points with the three-dimensional model, the concentration and temperature of the ternary gas mixture are obtained. Combining the two graphical algorithms with speed of sound reconstruction methods, a method for reconstructing gas concentration fields and temperature fields is developed. Finally, an AT system was built to complete the reconstruction of concentration and temperature fields of a binary gas mixture, verifying the feasibility and effectiveness of the proposed method.

The proposed method combines acoustic relaxation and acoustic tomography to provide a practical scheme for the joint inversion of gas concentration and temperature. Future work will focus on dynamic detection of gas state fields and detection in extreme environments.

## Figures and Tables

**Table 1:** Reconstruction errors for three distributions.

Distribution	$E_{\max}({\mathrm{CO}}_{2})$	$E_{\mathrm{mean}}({\mathrm{CO}}_{2})$	$E_{\mathrm{rms}}({\mathrm{CO}}_{2})$	$E_{\max}(T)$	$E_{\mathrm{mean}}(T)$	$E_{\mathrm{rms}}(T)$
Single-peak symmetric	26.77%	6.99%	11.58%	3.23%	0.53%	0.84%
Double-peak symmetric	16.31%	3.25%	5.31%	2.89%	0.43%	0.67%
Double-peak skewed	14.54%	3.70%	4.98%	2.59%	0.60%	0.78%

**Table 2:** Reconstruction errors for two distributions.

Distribution	$E_{\max}\left({\mathrm{CO}}_{2}\right)$	$E_{\mathrm{mean}}\left({\mathrm{CO}}_{2}\right)$	$E_{\mathrm{rms}}\left({\mathrm{CO}}_{2}\right)$	$E_{\max}\left({\mathrm{CH}}_{4}\right)$	$E_{\mathrm{mean}}\left({\mathrm{CH}}_{4}\right)$	$E_{\mathrm{rms}}\left({\mathrm{CH}}_{4}\right)$	$E_{\max}\left(T\right)$	$E_{\mathrm{mean}}\left(T\right)$	$E_{\mathrm{rms}}\left(T\right)$
Single-peak symmetric	20.25%	10.08%	10.52%	15.98%	5.61%	5.77%	5.63%	1.56%	1.68%
Double-peak symmetric	11.02%	4.43%	5.18%	13.80%	8.39%	8.92%	3.10%	1.20%	1.29%

**Table 3:** The measurement values at the test points of Experiment 1 and the reconstruction results obtained using the projection intersection method.

	Temperature Point 1	Temperature Point 2	Temperature Point 3	Temperature Point 4	Concentration Point
Reconstructed results	300.1 K	291.6 K	297.3 K	291.6 K	8.0%
Measured values	293.5 K	292.7 K	293.6 K	294.8 K	6.3%

**Table 4:** The measurement values at the test points of Experiment 2 and the reconstruction results obtained using the projection intersection method.

	Temperature Point 1	Temperature Point 2	Temperature Point 3	Temperature Point 4	Concentration Point
Reconstructed results	331.2 K	316.4 K	301.2 K	290.3 K	12.7%
Measured values	342.4 K	324.6 K	307.4 K	294.8 K	9.3%

**Table 5:** The measurement values at the test points of Experiment 3 and the reconstruction results obtained using the projection intersection method.

	Temperature Point 1	Temperature Point 2	Temperature Point 3	Temperature Point 4	Concentration Point
Reconstructed results	332.8 K	293.0 K	305.3 K	297.7 K	6.3%
Measured values	337.8K	300.4 K	298.3 K	296.7K	7.4%

## Data Availability

Data will be made available on request.
